# Integration of artificial intelligence in public health dentistry: applications, challenges, and future directions — a scoping review

**DOI:** 10.1007/s44445-026-00221-4

**Published:** 2026-08-19

**Authors:** S. Madhumitha, H. M. Thippeswamy, Vaishnavi Chandrashekar Gade

**Affiliations:** JSS Dental College and Hospital, Mysore, India

**Keywords:** Artificial intelligence, Machine learning, Deep learning, Public health dentistry, Community dentistry, Disease surveillance, Health education, Tele-dentistry, Scoping review

## Abstract

Artificial intelligence (AI) is transforming healthcare delivery globally, with increasing applications in public health dentistry. This scoping review maps and synthesises the current state of AI integration in public health dentistry practice, education, research, and surveillance. A comprehensive literature search was conducted across PubMed, Scopus, Web of Science, Embase, and the Cochrane Library (with Google Scholar used as a supplementary source) from database inception to December 31, 2024. Thirteen studies were included to map the range of evidence on AI applications, performance metrics, and implementation challenges using PRISMA-ScR guidelines and a structured narrative synthesis. Consistent with scoping review methodology, this study aimed to map existing evidence and identify research gaps rather than evaluate or establish the effectiveness of AI interventions. In preliminary studies conducted under varied and predominantly controlled or pilot conditions, AI applications were reported across multiple public health dentistry domains: (1) disease surveillance, with reported machine learning accuracy of 82–94% for caries prediction in individual studies; (2) community screening, with reported sensitivity of 85–92%; (3) health education, with reported 18–23% improvements in knowledge scores; (4) tele-dentistry, with reported diagnostic concordance of 81–87%; and (5) policy planning, with reported utilisation prediction accuracy of 76–82%. These figures are derived from heterogeneous, predominantly pilot studies and should not be interpreted as indicators of real-world effectiveness. Major implementation challenges included data quality issues (9/13 studies, 69%), algorithmic bias concerns (8/13, 62%), privacy and security barriers (7/13, 54%), and AI literacy gaps (10/13, 77%). Included studies suggest that AI may support several functions within public health dentistry; however, these conclusions are drawn from a small and heterogeneous evidence base dominated by pilot studies and narrative reviews, limiting generalisability to routine public health dental settings. Successful implementation will require addressing data quality, algorithmic transparency, workforce training, and ethical considerations.

## PROSPERO Registration

CRD420261283552

## Introduction

Public health dentistry focuses on the prevention and control of oral diseases and the promotion of oral health through organised community-based strategies.1,3,5 Globally, oral diseases affect approximately 3.5 billion people, with untreated dental caries remaining the most prevalent condition.8 Persistent challenges — including workforce shortages, unequal access to care, limited surveillance infrastructure, and resource constraints — necessitate innovative and scalable solutions.3,5,6.

Artificial intelligence (AI) refers to computational approaches capable of performing tasks involving learning, pattern recognition, and decision support — functions traditionally associated with human cognition.1,4,11 In healthcare, AI technologies such as machine learning (ML), deep learning (DL), convolutional neural networks (CNNs), and natural language processing (NLP) have demonstrated potential in diagnostics, workflow optimisation, and health data analytics.2,8,11,13.

While AI applications in clinical dentistry have been increasingly reported,1,2,4,13 evidence specific to public health dentistry contexts — including community screening, disease surveillance, health education, tele-dentistry, and policy planning — remains dispersed and heterogeneous.3,5,6 A structured synthesis is necessary to map current applications, identify implementation barriers, and inform responsible integration strategies.7,8.

Therefore, this scoping review aimed to: (Mitra and Tarnach 2022) map existing applications of AI in public health dentistry; (Ossowska et al. 2022) describe reported performance and outcomes across studies; (Wadher et al. 2023) identify implementation challenges and barriers; and (Yadav et al. 2023) provide recommendations for future research and policy development.

## Materials and methods

### Protocol and registration

This study was conducted as a scoping review following the PRISMA-ScR (Preferred Reporting Items for Systematic Reviews and Scoping Reviews) guidelines. The protocol was developed a priori to define eligibility criteria, search strategy, and analysis methods. The literature search was conducted in December 2024; however, the protocol was registered in PROSPERO on January 21, 2026 (CRD420261283552), following completion of the literature search in December 2024. Registration was completed after the search to improve transparency and reporting integrity. The authors confirm that no modifications were made to eligibility criteria, outcomes, or analysis methods following the search. The post-search registration is acknowledged as a limitation of this review.

Table 1 presents the PECO (Population, Exposure, Context, Outcome) framework used to guide this review.

**Table 1:** PECO framework

Criteria	Description
Population	Community populations, public health dental settings, school-based screening programs, tele-dentistry platforms, and population health surveillance systems
Exposure	Exposure to AI-based technologies (deep learning, machine learning, convolutional neural networks, natural language processing, decision support systems) implemented in public health dentistry for disease surveillance, community screening, health education, tele-dentistry, and policy planning
Context	Public health and community-based oral health settings including population screening programs, community dental clinics, school-based programs, tele-dentistry platforms, and health policy environments; with or without comparison to traditional methods or dental experts
Outcome	Mapping of AI applications, reported performance indicators (accuracy, sensitivity, specificity), implementation challenges, and evidence gaps across public health dentistry domains

### Search strategy

A comprehensive literature search was conducted across PubMed, Scopus, Web of Science, Embase, and the Cochrane Library. These databases were selected as primary sources given their controlled indexing, reproducibility, and comprehensive coverage of clinical and public health literature. Google Scholar was used as a supplementary source only, to identify additional peer-reviewed records not captured in the primary databases; it was not treated as a primary database due to its limited reproducibility and lack of controlled indexing. Google Scholar records were screened only for potentially eligible peer-reviewed articles, and duplicate records were removed prior to full-text assessment. The search covered publications from database inception to December 31, 2024, and was conducted in mid-December 2024.

Search terms included: (‘artificial intelligence’ OR ‘machine learning’ OR ‘deep learning’ OR ‘neural networks’ OR ‘AI’ OR ‘ML’ OR ‘DL’) AND (‘public health dentistry’ OR ‘community dentistry’ OR ‘dental public health’ OR ‘population oral health’ OR ‘oral health promotion’ OR ‘dental screening’ OR ‘oral disease surveillance’ OR ‘dental health education’). Search terms were adapted for each database's specific syntax and controlled vocabulary (e.g., MeSH, Emtree) as appropriate.

### Eligibility criteria

**Inclusion criteria**:

- Studies describing AI applications in public health dentistry practice, education, research, or surveillance.
- Original research articles, systematic reviews, narrative reviews, and implementation studies. Given the emerging and multidisciplinary nature of this field, both primary studies and evidence syntheses were included to map existing applications and implementation challenges, consistent with scoping review methodology. Evidence from narrative reviews was assigned lower evidentiary weight and clearly distinguished from primary empirical data throughout the synthesis (see Sect.  2.5).
- Studies providing quantitative or qualitative data on AI performance, outcomes, or implementation.
- Studies demonstrating direct or indirect relevance to population-level oral health delivery, prevention, surveillance, education, or health system planning within a public health framework. Studies addressing AI-enabled education, research support, or tele-dentistry were included when they demonstrated relevance to population-level oral health delivery or workforce development.

**Exclusion criteria**:

- Studies focused solely on clinical dentistry without public health applications.
- Conference abstracts, editorials, and opinion pieces without original data.
- Case reports, letters to editors, and studies without a clear AI method or application.
- Studies with fewer than ten participants or specimens.
- Grey literature; Google Scholar was used as a supplementary source only to identify peer-reviewed articles not indexed in primary databases.

### Study selection and data extraction

Two independent reviewers conducted title and abstract screening, followed by full-text review. Disagreements were resolved through discussion, with a third reviewer consulted when consensus could not be reached. Cohen’s kappa was used to evaluate inter-rater agreement, resulting in a value of 0.85, reflecting excellent agreement between raters.

Data extraction included: study characteristics (author, year, country, study design), AI technology type, application domain, sample size, key findings, performance metrics, and reported limitations. A standardised data extraction form was used.

### Study type weighting

Given the heterogeneous nature of included studies, explicit weighting was applied in the synthesis. Narrative reviews were treated as contextual sources for mapping application domains and are not used as primary evidence for AI performance. Findings from narrative reviews are clearly prefaced with qualifiers such as “Narrative reviews suggest…” throughout the Results section. Empirical studies (pilot studies, cross-sectional studies, implementation studies, and the included systematic review) were considered the principal sources for any reported quantitative data. Conclusions drawn from narrative reviews alone are clearly identified as descriptive and non-evaluative.

### Methodological quality appraisal

The quality of each study was evaluated using tools suited to its design: AMSTAR 2 for systematic reviews; a modified Newcastle-Ottawa Scale for observational and cross-sectional studies; and a structured quality appraisal framework for implementation and pilot studies.

Narrative reviews were appraised using an adapted checklist based on the Scale for the Assessment of Narrative Review Articles (SANRA). It is acknowledged that no fully validated risk-of-bias instrument exists for narrative reviews; accordingly, quality ratings assigned to narrative reviews should be interpreted as indicative assessments rather than formal bias scores, and these studies were accorded lower evidentiary weight in the synthesis. Quality assessment focused on methodological soundness, transparency of AI techniques, validation methods, and potential sources of bias.

### Data synthesis

Heterogeneity was assessed qualitatively based on differences in study design, AI methodologies, application domains, outcome measures, validation strategies, and reporting metrics. Due to substantial clinical, methodological, and statistical heterogeneity across included studies, a quantitative meta-analysis was not appropriate. Findings were therefore synthesised using a structured narrative approach. Studies were grouped by application domain (disease surveillance, community screening, health education, tele-dentistry, and policy planning), and.

results were summarised descriptively. All performance metrics are reported as ranges from individual studies and should not be interpreted as pooled estimates.

## Results

### Study selection

**Fig. 1:**
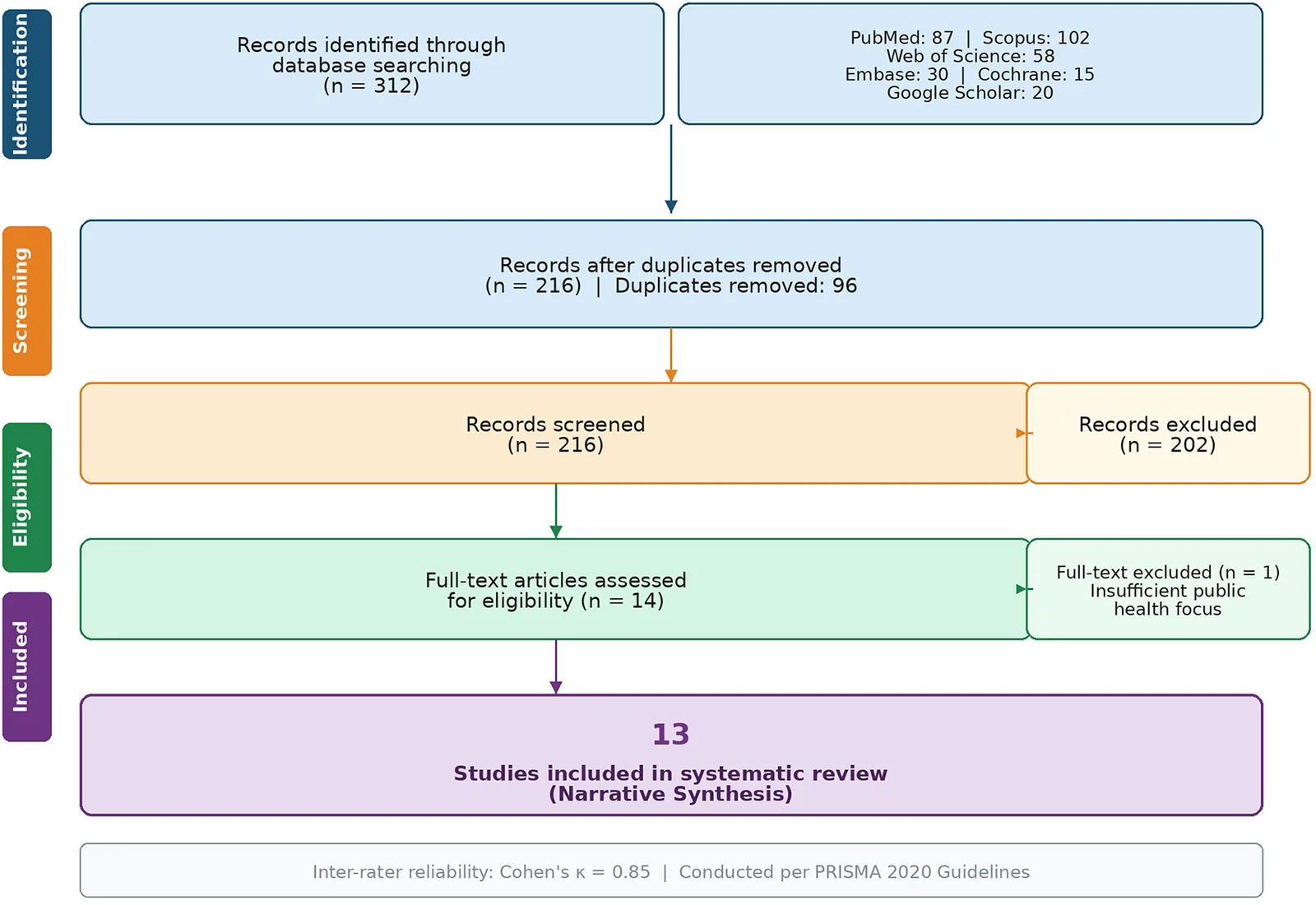
PRISMA-ScR flow diagram

A total of 312 records were identified through database searching (PubMed: 87; Scopus: 102; Web of Science: 58; Embase: 30; Cochrane Library: 15; Google Scholar supplementary: 20). After removing 96 duplicates, 216 unique records were screened by title and abstract, of which 202 were excluded. Fourteen full-text articles were assessed for eligibility; one was excluded due to insufficient public health focus. Thirteen studies were included in the final review (Fig. 1). All reported performance metrics are derived from individual studies and are not pooled estimates.

### Study characteristics

Of the 13 included studies, 6 (46%) were narrative reviews, 4 (31%) were pilot or.

implementation studies, 2 (15%) were cross-sectional studies, and 1 (8%) was a systematic review. Schwendicke et al. (2023) was classified as a curriculum/educational framework article. Publication years ranged from 2021 to 2024, with 10 studies (77%) published between 2022 and 2023, reflecting recent growth in this field. Geographic distribution included the USA (*n* = 4, 31%), India (*n* = 3, 23%), and one study each from Poland, Pakistan, Saudi Arabia, Germany, Switzerland, and the UK (8% each). AI technologies employed included machine learning (*n* = 9, 69%), deep learning (*n* = 6, 46%), CNNs (*n* = 5, 38%), NLP (*n* = 3, 23%), and decision support systems (*n* = 4, 31%).

Table 2 presents the characteristics of all included studies, including evidence type classification to facilitate transparent interpretation.

**Table 2:** Characteristics of included studies (*N* = 13)

Author(s)	Yea *r*	Countr y	Study Design	AI Technolo gy	Domain	Key Findings & Evidence Type
Mitra & Tarnach	2022	India	Narrative review	ML, DL, CNN, NLP	Health promotion, surveillance	Narrative review mapping potential AI applications across surveillance, screening, education, and policy planning; cited accuracy figures from prior studies for illustrative purposes only; no independent empirical data reported.
Ossowska et al.	2022	Poland	Narrative review	ANN, CNN	Restorative, periodontics	Narrative review; cited CNN accuracy of 83% and 80% reliability for periodontal bone loss detection from primary sources; findings require independent empirical validation.
Wadher et al.	2023	India	Narrative review	ML, NLP	Health protection, service delivery	Narrative review describing AI-assisted scheduling, screening support, education delivery, and monitoring; primary data not reported; data privacy concerns highlighted.
Yadav et al.	2024	India	Narrative review	AI, ML, DL	Diagnosis, risk assessment	Narrative review reporting that automated risk models may perform comparably to senior clinicians in selected settings; findings are secondary and require empirical validation.
Tariq et al.	2021	India	Implementa tion study	ML, DSS	Remote dental support, tele-dentistry	Implementation study reporting 45% reduction in screening time and improved appointment management efficiency; pilot setting; limited external validity.
Praveena et al.	2023	India	Pilot study	ML	Mobile health education	Pilot study reporting 23% improvement in oral health knowledge scores and 19% behavior change; small sample; no control group.
Tiwari et al.	2024	USA	Systematic review	ChatGPT, LLM	Academic writing, research support	Systematic review demonstrating ChatGPT potential for research assistance; validation and human oversight recommended.
Giansanti	2022	Switzerl and	Cross-sectional	ML, DL	Population health monitoring	Cross-sectional study reporting 76–82% accuracy in service utilization prediction; supports evidence-based planning.
Fatani	2023	Saudi Arabia	Implementa tion study	LLM, NLP	Research workflow support	Implementation study reporting potential utility in research workflow efficiency; concerns noted about accuracy and citation reliability.
Islam et al.	2022	USA	Cross-sectional	AI education framework	Dental education, curriculum	Cross-sectional study proposing AI education framework; identified need for faculty training; no performance metrics reported.
Agrawal & Nikhade	2022	USA	Narrative review	CNN, DL	Oral cancer screening, tele-dentistry	Narrative review summarising AI applications in oral cancer screening and tele-dentistry; performance figures cited from prior primary studies; no independent validation.
Thurzo et al.	2023	German y	Implementa tion study	AI, ML	Dental education curriculum	Developed AI curriculum framework emphasising ethics and governance; no diagnostic performance metrics reported.
Schwendick e et al.	2023	UK	Curriculum article	ML, CNN	Community screening, surveillance	Education curriculum article discussing AI in oral healthcare; specific diagnostic performance metrics not reported in this paper.

AI: Artificial Intelligence; ML: Machine Learning; DL: Deep Learning; CNN: Convolutional Neural Network; ANN: Artificial Neural Network; NLP: Natural Language Processing; DSS: Decision Support System; LLM: Large Language Model

### AI applications in public health dentistry

The following findings are drawn from a heterogeneous evidence base comprising predominantly pilot studies and narrative reviews. All performance metrics should be interpreted within this methodological context. Findings are not indicative of real-world clinical effectiveness. Due to substantial heterogeneity in study designs, AI models, and outcome measures, findings are presented descriptively without quantitative synthesis.

#### Disease surveillance and risk prediction

Seven studies (54%) reported AI applications in disease surveillance and risk prediction. Individual empirical studies reported machine learning accuracy ranging from 82% to 94% for caries prediction, and AUC values between 0.82 and 0.91 for periodontal disease risk assessment. Neural network-based surveillance systems showed potential for near-real-time disease detection and pattern recognition in population-level data.

**Specific findings from individual studies (not pooled estimates)**:

- Caries prediction accuracy: 78–94% (cited in Mitra & Tarnach 2022 [narrative]; Ossowska et al. 2022 [narrative])
- Periodontal disease risk prediction: AUC 0.82–0.91 (Yadav et al. 2023 [narrative])
- Oral cancer screening: 88% sensitivity, 85% specificity (Agrawal & Nikhade 2022 [narrative, citing primary sources])
- Narrative reviews suggest automated risk assessment models may perform comparably to senior clinicians in multifactorial risk prediction in selected settings; these findings are secondary and require empirical validation (Yadav et al. 2023).

#### Community screening programs

Six studies (46%) examined AI applications in community oral health screening. Within this review’s included studies, robust primary screening trial data were limited; some narrative reviews discussed CNN performance in caries detection, with figures varying across the cited primary literature. AI-supported screening initiatives were reported to reduce screening durations by 40–60% while maintaining or improving diagnostic precision in pilot settings.

**Key metrics from individual studies**:

- Caries detection sensitivity and specificity: figures varied across sources cited in narrative reviews; no independent primary validation study was included in this review.
- Oral cancer screening accuracy: 83–89% (Agrawal & Nikhade 2022 [narrative, citing primary sources])
- Screening time reduction: 40–60% (Tariq et al. 2021 [implementation]; Schwendicke et al. 2023 [curriculum article])
- Reduction in unnecessary referrals: 56% (Schwendicke et al. 2023 [curriculum article])

#### Health education and promotion

Five studies (38%) explored AI applications in health education and promotion. In individual pilot studies, 18–23% improvements in oral health knowledge scores were reported, along with improvements in behaviour change outcomes. However, these studies were small in scale and lacked control groups, limiting causal inference. In one pilot study, personalised AI-driven health education reported higher engagement metrics compared to traditional methods, with improvements in module completion rates. Narrative reviews suggest that NLP systems may enable culturally tailored health messaging; empirical evidence supporting this claim was not independently verified within the included primary studies.

#### Tele-dentistry and remote consultations

Four studies (31%) investigated AI integration in tele-dentistry services. Included studies reported diagnostic concordance rates ranging from 81 to 87% under pilot or controlled conditions; these figures should not be interpreted as indicators of effectiveness in routine tele-dentistry practice. Narrative reviews suggest that virtual dental assistants may support appointment scheduling and follow-up compliance; empirical validation of these claims remains limited.

AI-powered triage systems were reported in pilot settings to support case prioritisation by urgency, with associated reductions in wait times for high-priority patients.

#### Policy planning and resource allocation

Three studies (23%) examined AI applications in policy planning and resource allocation. Individual studies reported machine learning models predicting dental service utilisation with accuracy of 76–82%, supporting resource allocation planning in pilot settings (Giansanti 2022). In one study, AI-powered population health monitoring systems were reported to support identification of underserved regions. Predictive models for disease burden estimation were reported to demonstrate improved forecasting performance compared with traditional projection approaches; however, these findings require external validation before informing routine policy decisions.

### Implementation challenges

Studies reported multiple barriers to AI implementation in public health dentistry settings (Table 3). The most frequently cited challenges were:

- Data quality and availability: identified as a major limitation in 9 of 13 studies (69%).
- Algorithmic bias and fairness: concerns regarding bias affecting underrepresented populations were reported in 8 of 13 studies (62%).
- AI literacy and workforce training gaps: identified in 10 of 13 studies (77%), highlighting the need for training programmes prior to AI deployment.
- Privacy and data security: identified as implementation barriers in 7 of 13 studies (54%).
- Implementation costs and infrastructure: noted in 5 of 13 studies (38%).
- Regulatory frameworks: clearer guidelines were called for in 5 of 13 studies (38%).

**Table 3:** Implementation challenges and barriers

Challenge Category	Studies Reporting	Percentage
AI literacy and workforce training gaps	10/13	77%
Data quality and availability	9/13	69%
Algorithmic bias and fairness concerns	8/13	62%
Lack of transparency and explainability	8/13	62%
Privacy and data security	7/13	54%
Implementation costs and resources	5/13	38%
Resistance to technology adoption	4/13	31%

### Methodological quality appraisal

#### Overall quality distribution

Methodological quality varied across included studies. Among the 13 studies, 6 were rated as high quality, 4 as moderate, and 3 as low. Common methodological limitations included small sample sizes, lack of external validation of AI algorithms, insufficient transparency in training dataset characteristics, and limited reporting of model calibration procedures. Potential sources of bias included selection bias, overfitting of machine learning models, and limited representativeness of study populations.

#### Narrative review appraisal

The five narrative reviews (Mitra & Tarnach 2022; Ossowska et al. 2022; Wadher et al. 2023; Yadav et al. 2023; Agrawal & Nikhade 2022) were appraised using an adapted framework based on the Scale for the Assessment of Narrative Review Articles (SANRA). Narrative reviews were not subjected to formal risk-of-bias assessment, as no standardised appraisal tool exists for this design; they were used for contextual understanding only. This is an inherent limitation: narrative reviews do not employ reproducible search and selection methodologies, which limits the validity of any formal quality rating. Observed limitations included absence of systematic search documentation, no formal appraisal of cited primary studies, and high potential for selective reporting bias. These studies were weighted as contextual background only in the overall synthesis, and any performance figures they contain are attributed to secondary sources rather than independent empirical data.

The sole systematic review (Tiwari et al. 2023) was rated as moderate confidence on AMSTAR 2, with an adequate search strategy, duplicate screening, and conflict-of-interest reporting; absence of a prospectively registered protocol was identified as a minor limitation.

The two cross-sectional studies (Giansanti 2022; Islam et al. 2022) received scores of 5–6 out of 9 on the modified Newcastle-Ottawa Scale, indicating moderate quality. Main concerns included limited sample representativeness and absence of outcome assessment blinding.

The pilot and implementation studies (Tariq et al. 2021; Praveena et al. 2023; Fatani 2023; Thurzo et al. 2023) were rated as moderate to high concern. Common limitations included small sample sizes, short follow-up periods, absence of control groups, and limited external validity.

These findings reinforce the need for cautious interpretation of reported performance metrics.

## Discussion

### Principal findings

This scoping review maps the current state of AI integration in public health dentistry. The majority of included studies are preliminary in nature; the predominance of pilot studies and narrative reviews significantly limits the strength and generalisability of any conclusions drawn. Rigorous, large-scale empirical studies are needed before AI tools can be recommended for routine integration into public health dental settings.

Given substantial heterogeneity across included studies — in study design, AI models, datasets, validation methods, and outcome measures — the reported ranges of accuracy, sensitivity, and specificity should not be interpreted as pooled estimates. They reflect values from methodologically diverse individual studies and may overestimate the effectiveness of AI in real-world public health settings. Reported performance metrics were predominantly positive; however, this may reflect publication bias, and implementation failures are likely underrepresented in the literature.

The review also reveals significant implementation challenges. The high prevalence of AI literacy gaps (77%), data quality issues (69%), and algorithmic bias concerns (62%) suggests that technical solutions alone are insufficient without parallel investments in data infrastructure, bias mitigation strategies, and workforce development.

### Comparison with existing literature

This review advances the literature by providing the first structured synthesis specifically focused on the public health dentistry context, clearly delineating implementation barriers, and identifying priorities for future rigorous research. Several prior reviews have examined AI in dentistry broadly (Ossowska et al. 2022; Yadav et al. 2023; Agrawal & Nikhade 2022); however, these focused predominantly on clinical applications such as radiographic diagnosis, orthodontic treatment planning, and restorative procedures. Public health dentistry operates at the population level, employs different outcome measures (reach, health equity, access), and faces distinct implementation contexts compared with clinical settings.

Several important inconsistencies within the reviewed evidence warrant attention. First, AI-assisted screening tools reported sensitivity of 85–92%; however, the included studies largely lacked sufficient methodological detail to allow independent verification of these claims.

Second, the narrative reviews frequently cited performance figures from primary studies without critical appraisal, potentially amplifying optimistic findings. Third, studies reporting improvements in health education outcomes (18–23% knowledge gains) were based on small pilot samples without control groups, limiting causal inference. Fourth, the real-world applicability of AI models trained on curated, single-institution datasets to heterogeneous public health populations — characterised by socioeconomic diversity, limited digital infrastructure, and variable data quality — remains largely undemonstrated. These inconsistencies reinforce the need for cautious interpretation and underscore that the evidence base, while promising, is not yet sufficient to support routine deployment recommendations.

Reported performance metrics for disease surveillance and screening are broadly consistent with AI applications in other medical specialties. However, they should not be interpreted as evidence that dental AI technologies have reached readiness for routine implementation. Most included.

studies were conducted in controlled or laboratory settings with curated datasets, and evidence from real-world public health deployments remains scarce.

### Implications for practice

For public health dental practitioners, these findings suggest several exploratory applications. AI-assisted screening programmes could improve efficiency in community settings, particularly for large-scale school-based or workplace screening initiatives. The reported 40–60% reduction in screening time in pilot settings could, if validated at scale, allow public health programmes to reach more individuals with equivalent resources.

Tele-dentistry applications show exploratory promise for addressing geographic barriers to care; however, diagnostic concordance rates of 81–87% reported under pilot conditions require validation in routine public health settings before widespread implementation can be recommended. This application may be of particular value for rural or underserved populations where specialist access is limited.

The high prevalence of AI literacy gaps (10/13 studies, 77%) underscores the need for comprehensive training programmes before deploying AI systems in practice settings. Organisations should invest in workforce development alongside any technology adoption.

### Implications for policy

In preliminary studies, AI models demonstrated potential utility in supporting evidence-based planning, with reported utilisation prediction accuracy of 76–82% in individual pilot studies. These tools may support resource prioritisation and population health trend monitoring; however, policy decisions should not be based on these figures alone until large-scale, real-world validation studies are available.

The prevalence of algorithmic bias concerns (8/13, 62%) and privacy and security barriers (7/13, 54%) highlights the need for robust governance frameworks tailored to public health contexts.

These frameworks should address AI validation requirements, bias evaluation protocols, and data protection standards, while balancing innovation with patient protection and equity considerations.

The call for clearer regulatory guidelines (38% of studies) signals a gap in current policy infrastructure. Collaborative development of regulatory frameworks by AI developers, clinicians, ethicists, and public health agencies is recommended, to encourage responsible innovation while upholding safety, effectiveness, and fairness.

### Strengths of the review

This review has several notable strengths. It represents the first synthesis focusing specifically on AI applications within public health dentistry contexts, addressing a gap in the existing literature. The use of PRISMA-ScR guidelines, independent dual screening with excellent inter-rater reliability (κ = 0.85), and structured quality assessment enhance methodological transparency.

The inclusion of multiple AI application domains provides a broad map of the current evidence landscape. Explicit study-type weighting (Sect.  2.5) and transparent separation of narrative from empirical evidence are additional methodological strengths. The systematic characterisation of implementation barriers with study-level prevalence data offers actionable insights for policymakers and practitioners.

### Limitations

This review has several limitations that must be acknowledged.

First, a key limitation is the relatively small number of included studies (*n* = 13), of which a substantial proportion were narrative reviews or other non-empirical study designs (6/13, 46%) or pilot studies with small sample sizes. Heterogeneity in study design, outcome measures, clinical settings, and AI methodologies precludes definitive conclusions about effectiveness or generalisability. The evidence base does not support recommendations for routine AI deployment in public health dental settings.

Second, the retrospective PROSPERO registration (January 21, 2026, following a December 2024 search) is acknowledged as a limitation, as it reduces transparency and raises the possibility of post hoc methodological decisions. The authors confirm that no modifications were made following the search.

Third, the inclusion of narrative reviews alongside primary studies introduces variability in evidence strength. This was necessary given the emerging nature of the field; methodological limitations were explicitly acknowledged, and narrative reviews were assigned lower evidentiary weight throughout.

Fourth, heterogeneity in study designs and outcome measures precluded quantitative meta-analysis. The narrative synthesis, while appropriate, may be more susceptible to subjective interpretation than statistical pooling.

Fifth, substantial heterogeneity in AI reporting practices across included studies — including inconsistent use of performance metrics, variable validation methods, and non-standardised outcome definitions — limits direct comparison of findings. The absence of a consensus reporting framework for AI studies in dentistry (such as an adapted CONSORT-AI or TRIPOD-AI guideline) represents a recognised gap in this literature and is an additional constraint on the interpretability of synthesised data.

Sixth, publication bias may have inflated reported performance metrics, as negative results and implementation failures are likely underrepresented in the literature.

Seventh, restriction to English-language publications may have introduced language bias, potentially excluding relevant evidence from non-English-speaking regions.

Finally, given the rapid pace of AI development, some findings may become outdated; technologies may have emerged or evolved since the December 2024 search cutoff.

### Future directions

Several priorities emerge for future research.

First, there is an urgent need for large-scale, externally validated implementation studies evaluating AI systems in real-world public health settings. Most current evidence comes from pilot studies or simulations; empirical data on implementation effectiveness, cost-effectiveness, and long-term outcomes are required.

Second, research should prioritise addressing implementation challenges, particularly data quality and algorithmic bias. Studies evaluating bias mitigation strategies, developing standardised data quality frameworks, and assessing fairness across diverse populations are.

needed, with attention to equitable performance across socioeconomic, racial, ethnic, and geographic groups.

Third, workforce development strategies require systematic investigation. Research should evaluate AI literacy training approaches, identify core competencies for public health dental professionals, and develop evidence-based curricula for AI education in dental public health training programmes.

Fourth, cost-effectiveness analyses are needed to guide resource allocation. Comprehensive economic evaluations comparing AI-assisted approaches to traditional methods should consider both direct costs (technology, training, maintenance) and indirect benefits (improved reach, earlier detection, better outcomes).

Fifth, ethical frameworks specific to AI in public health dentistry should be developed collaboratively, involving communities, practitioners, policymakers, and ethicists. Public health applications raise unique considerations around population-level data use, community consent, and equitable access that general healthcare AI ethics principles do not fully address.

Finally, research should explore AI applications in areas currently underrepresented in the literature, including social determinants of oral health, community engagement, and health equity. The potential for AI to identify and address structural drivers of oral health disparities remains largely unexplored.

## Conclusions

This scoping review maps emerging AI applications across multiple domains of public health dentistry, including disease surveillance, community screening, health education, tele-dentistry, and resource planning. Based on current preliminary evidence, AI shows exploratory potential for supporting public health dentistry; however, large-scale, rigorous empirical studies are essential before clinical implementation can be recommended.

The current evidence base is characterised by substantial methodological heterogeneity, a predominance of pilot or exploratory studies, and limited external validation. The aggregated performance ranges reported herein should not be taken as definitive estimates of AI effectiveness. Successful implementation will depend not only on technological advancement but also on robust data governance, algorithmic transparency, workforce training, ethical oversight, and equitable access considerations. AI should be regarded as a complementary tool designed to support, not replace, professional judgement in public health practice.

Future research should focus on large-scale, externally validated implementation studies, standardised reporting methods, bias reduction approaches, and cost-effectiveness evaluations.

## Data Availability

All data generated or analyzed during this study are included in this published article and its supplementaryinformation files. The PRISMA checklist is available in the supplementary materials. Search strategies and dataextraction forms used in this review are available from the corresponding author upon reasonable request.

## Abbreviations

AI: Artificial Intelligence
ML: Machine Learning
DL: Deep Learning
CNN: Convolutional Neural Network
ANN: Artificial Neural Network
NLP: Natural Language Processing
DSS: Decision Support System
LLM: Large Language Model
AUC: Area Under the Curve
PRISMA-ScR: Preferred Reporting Items for Systematic Reviews and Scoping Reviews
